# Rapidly progressive dementia: An eight year (2008–2016) retrospective study

**DOI:** 10.1371/journal.pone.0189832

**Published:** 2018-01-18

**Authors:** Patil Anuja, Vishnu Venugopalan, Naheed Darakhshan, Pandit Awadh, Vinny Wilson, Goyal Manoj, Modi Manish, Lal Vivek

**Affiliations:** 1 Department of Neurology, Postgraduate Institute of Medical Education and Research, Sector 12, Chandigarh, India; 2 Department of Neurology, All India Institute of Medical Sciences, New Delhi, India; Nathan S Kline Institute, UNITED STATES

## Abstract

**Background and purpose:**

Rapidly progressive dementia (RPD) is an emergency in cognitive neurology, defined as cognitive impairment affecting the daily living activities developed over less than 1 year. This study investigated the profile of patients with rapidly progressive dementia at first presentation.

**Methods:**

Retrospective case analysis was done in 187 patients with rapidly progressive dementia who presented to the Postgraduate Institute of Medical Education and Research, Chandigarh, India from January 2008 to August 2016. Patients were divided into three groups: (1) Reversible (treatable) secondary dementia group, (2) Prion dementia group (sporadic Creutzfeldt-Jakob disease), (3) Non-prion Neurodegenerative and vascular dementias (primary neurodegenerative and vascular dementia). Cases presenting with delirium secondary to metabolic, drug induced or septic causes and those with signs of meningitis were excluded.

**Results:**

Secondary reversible causes formed the most common cause for RPD with immune mediated encephalitides, neoplastic and infectious disorders as the leading causes. The patients in this series had an younger onset of RPD. Infections presenting with RPD accounted for the most common cause in our series (39%) with SSPE (41%) as the leading cause followed by neurosyphilis (17.9%) and progressive multifocal leukoencephalopathy (15.3%). Immune mediated dementias formed the second most common (18.1%) etiologic cause for RPD. The neurodegenerative dementias were third common cause for RPD in our series. Neoplastic disorders and immune mediated presented early (< 6 months) while neurodegenerative disorders presented later (> 6 months).

**Conclusions:**

Rapidly progressive dementia is an emergency in cognitive neurology with potentially treatable or reversible causes that should be sought for diligently.

## Introduction

Degenerative dementias develop insidiously over years and are often diagnosed in late stages of the illness. Rapidly progressive dementias (RPD) on the other hand include a myriad of conditions that functionally disable an individual within a span of few days to years.[1–3] These are therefore recognized earlier and provide an opportunity for intervention.

With the advent of newer serological and imaging aids, a genre of immune mediated dementias is evolving as a treatable entity, even though prion diseases and neurodegenerative disorders have been implicated as the common etiologies presenting with RPD in most of the series published. Infectious and nutritional causes of dementias may remain undiagnosed unless actively sought for. Dementia demands exhaustive work up to determine the cause which may span across the whole spectrum of diagnostic modalities ranging from routine serological investigations and imaging to invasive tests involving brain biopsies. Thus, a systematic and stratified approach for confirming the diagnosis and etiology is important.

This is a retrospective study of patients with RPD at presentation in a tertiary care centre, emphasizing the importance of simple routine tests that aid in diagnosing the etiology especially in developing countries.

## Methods

We screened the medical records of patients admitted at a tertiary care center in north India (Postgraduate Institute of Medical Education and Research) from January 2008 to August 2016. The study was approved by the Postgraduate Institute of Medical Education and Research institute ethics committee. All data were fully anonymized prior to analyses of the current study. Patients with rapidly progressive or young onset cognitive decline with suspected secondary causes for dementia or those with fluctuating course of symptoms were preferentially admitted in our centre. Dementia was defined using the Diagnostic and Statistical manual of mental disorders- 5 (DSM-V) criteria [3] for major neurocognitive impairment (decline in one or more cognitive domains with functional impairment). Those patients presenting within one year of symptom onset were selected for evaluation. Cases presenting with delirium (defined according to DSM-V as fluctuating disturbance in attention and awareness developing over short period of time) secondary to metabolic or septic causes or with signs of meningitis were excluded. Also patients whose symptoms could be explained due to drug intoxication or abuse or adverse effects were excluded from the study. Those with diagnosed psychiatric disorders which could explain the symptoms during presentation were excluded.

Alzheimer’s dementia was defined as per the National Institute on Aging–Alzheimer’s association [4] criteria.[4] Vascular dementia was defined according to the National institute of neurological and communicative disorders and stroke Association criteria.[5] Dementia with Lewy bodies (DLBD) was diagnosed using the consensus guidelines for the clinical diagnosis of DLB.[6] Patients with frontotemporal lobar degeneration (FTLD) subtype -behavioural variant FTD was diagnosed using the international consensus criteria.[7] Semantic dementia variant of FTD/primary progressive aphasia was diagnosed according to the consensus criteria[8].

Cases suspected of autoimmune encephalistis were screened using Graus et al [9] criteria with subacute onset working memory deficits, seizures or psychiatric disturbances, bilateral T2/FLAIR hyperintensities or PET showing increased upatake in medial temporal lobes, CSF pleocytosis or EEG showing epileptic or slow wave activity over temporal lobes after exclusion of alternative causes. Antibodies against cell surface, synaptic or onconeural proteins were available for selected cases due to financial constraints, cases with positive results were diagnosed as definite autoimmune encephalitis while those with negative results or non-availability of antibody testing were diagnosed as per the Graus criteria [9].

Patients with suspected subacute sclerosing panencephalitis (SSPE) were diagnosed using Dyken et al [10] criteria. Cases with progressive and subacute cognitive deterioration with typical signs like myoclonus were further evaluated to rule out other differential disorders. EEG was performed in all cases with the typical periodic high voltage discharges at 4–6 seconds considered diagnostic. Brain biopsy or autopsy evaluation was not available for any of the cases. Anti-measles antibody testing for CSF and serum samples was available in selected cases as per the records, with diagnosis of probable SSPE in these cases as per the Dyken criteria [10].

Patients presenting with rapid cognitive decline due psychiatric disturbances like bipolar disorders and major depressive disorders were diagnosed using DSM-V [3] after ruling out other possible secondary and neurodegenerative disorders. Those with prior history of psychiatric disorders were excluded from the study.

Cases suspected with Creutzfeldt-Jakob disease were diagnosed using European criteria for 2009 sporadic Creutzfeldt-Jakob disease (sCJD).[11] Patients suspected of primary CNS vasculitis (PACNS) were diagnosed based on Calabrese and Mallek criteria [12] and subjected to vascular imaging with either CT/MRI angiography. Digital Substraction angiography (DSA) and/or biopsy were available in selected cases. Systemic vasculitis work up was done in all patients. Brain imaging in the form of magnetic resonance imaging (MRI) or computed tomography (CT) scan was obtained in all cases with EEG and CSF routine analysis in selected cases. Patients with suspected neoplastic disorders underwent brain biopsies.

Rapidly progressive dementia was defined where cognitive impairment affecting the daily living activities developed over less than 1 year. There are no set criteria or definition for RPD, which has been described to range over few months to 4 years. In our study we selected patients with symptom onset of <1year duration, as in previous series of RPD[13,14,15].

Clinical details were obtained from the hospital records. Demographic features, co-morbidities, presenting cognitive domains, associated neurological deficits were compared. Available data regarding biochemical, hemogram, thyroid function tests, serological work up for suspected connective tissue disorders, serum and CSF venereal disease research laboratory (VDRL) test, viral markers including Human immunodeficiency virus (HIV), hepatitis B (HBV)and C (HCV) screen, other CSF investigations and imaging reports including brain MRI, CT chest-abdomen and positron emission tomography (PET) scan were assessed. Autoimmune antibody panel testing was available in selected cases.

Age of onset was defined as per the age at which symptom onset was noticed by the caregivers. Symptom duration was defined in terms of days-months since onset of cognitive impairment till presentation to our health care facility. The major cognitive domains involved at the onset were noted. Associated other neurological deficits were compared. Higher mental function evaluation was recorded for all cases including mini mental status score at presentation and detailed lobar function tests (forward and backward digit span, clock drawing, category generation, graphic and motor Luria tests, calculation, apraxia testing, visual memory, paired learning and language assessment).

Patients were divided into three groups:

Group 1: Reversible (treatable) secondary dementia group

Reversible secondary dementia included all treatable causes like nutritional and metabolic causes, immune-mediated dementias and encephalopathies, infectious causes, neoplastic and metastatic disorders, psychiatric conditions (pseudo-dementias), demyelinating disorders and CNS vasculitis presenting with cognitive impairment.

Group 2: Prion dementia group (sCJD)

Group 3: Non-prion Neurodegenerative and vascular dementias (primary neurodegenerative and vascular dementia)

## Statistical analysis

The demographic and clinical profile was analyzed as per the categorization. The age of onset, symptom duration and MMSE scores at presentation were compared between the groups using ANOVA and post hoc tests.

## Results

**Fig 1:**
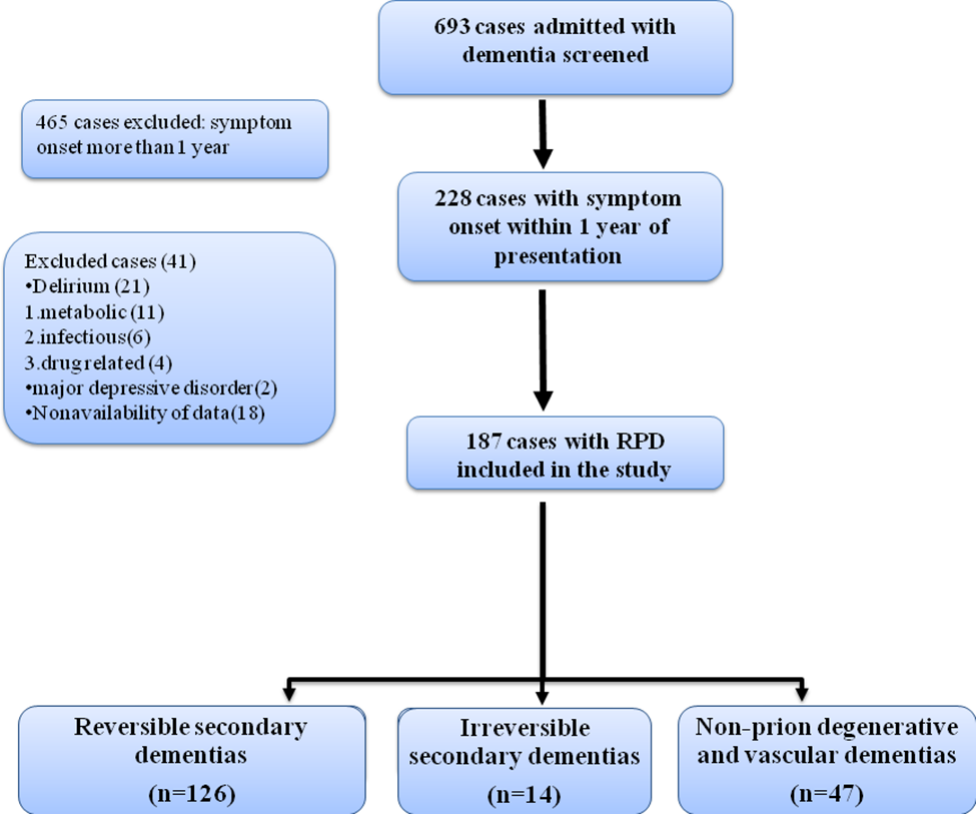
Flow chart of study design.

We screened 693 case records of patients admitted under neurology services with dementia from Jan 2008 to Aug 2016. Of these 465 cases were excluded due to time from symptom onset to dementia of more than a year. Of 228 cases with symptom onset one year or less, 23 cases were excluded due to presence of delirium, and 18 for the non-availability of records. 187 cases with RPD were included in study (Fig 1).

## Demographic profile of the study population

In the total study population 122 (65.2%) were males. Mean age at presentation was 49.3 ±18.21 years (13–84 years) with median of 50 years. The median duration of symptom onset was 4 months (mean ±SD = 5.28±4.1).

## Etiological categories

The clinical profile of patients across to various etiological subgroups is shown in Table 1.

**Table 1:** Clinical and demographic profile of patients as per the etiologic subgroups.

Sr no.	Etiological subgroup	No (n)	Mean age±SD years	Median age (IQR) years	Males (%)	duration in months Mean ± SD (median)	Average MMSE score (mean±SD)
1	Infectious disorders	39	32.4±16.8	25(20–42)	26(81.2%)	5.9±3.9(4)	18.14±6.09
2	Immune mediated encephalitis/encephalopathy	34	54.5±16.9	55 (45,69.2)	16(50%)	3.9±3.8 (2)	18.15±7.07
3	Neurodegenerative disorders	27	56.2±13.3	58(43.5–65.5)	21(84%)	9.2±3.9(12)	18.6±5.83
4	Neoplastic or metastatic disorders	25	56.17±14.1	58 (47.5–60.5)	15(65.2%)	3.04±2.6 (1)	18.8±7.71
5	Vascular cognitive imparment and other cerebrovascular events:	18	62.3±13.7	59 (50.7–78)	13(81.2%)	6.7±3.4(6)	11.4±3.5
6	Prion diseases	14	57.7±9.6	58(47.7–67)	7(50%)	3.8±3.3(3)	20.5±2.06
7	PACNS:	10	35±9.9	35.5(30.5–41)	8(80%)	5.9±4.4(5.5)	22.3±6.28
8	Nutritional and metabolic disorders	7	42.14±15.1	50 (25–54)	5(83.3%)	6.3± 4.8(8)	18.25±5.9
9	Demyelinating disorders:	6	38.5±17.7	39.5(21–51.5)	3(50%)	3.33±3.5(2.25)	24.6±2.64
10	Psychiatric Conditions:	5	56.8±13.1	64(42.5–67.5)	1(20%)	5±4.3(4)	28.2±1.3
11	Mixed / undetermined dementia	2	53.3±20.9	53	6(60%)	5.2±4.02(4.5)	22±2

SD: standard deviation, IQR: interquartile range, MMSE: mini mental status examination, PACNS: primary CNS vasculitis

Infections represented the largest proportion of our RPD patients; SSPE, HIV with Progressive Multifocal Leukoencephalopathy (PML) and neurosyphilis were leading causes. Table 2 shows characteristics of patients with infectious causes presenting with RPD.

**Table 2:** Infectious causes presenting with rapidly progressive dementia.

Sr. no	Diagnosis:	No. of patients:	Symptom duration:	MMSE (n±SD)	Investigations:
1	HSV encephalitis	1	15days	8	CSF HSV PCR: positive
2	Tubercular meningitis	2	5.5months	19.12	CSF: normal; communicating HCP with ring enhancing granulomas in parietal lobe.
3	Cryptococcal meningitis	1	4months	21	Cryptococcal antigen titre-1:64
4	TBM+ Cryptococcal meningitis	1	12months	17	CSF proteins:110, sugar:10, ADA:16, cryptococcal culture +; BAL: AFB2+
5	Neurocysticercosis	3	3.3months	17	Not done: 2; Normal: 1
6	SSPE	17	3.94months	17.5±7.7	Raised CSF anti-measles antibody titre: 9; normal titre:1; not done: 6
7	HIV dementia	1	3months	24	CSF: not done
8	HIV + PMLE	6	4.33months	15±1.41	Mean CD4 count:95;
9	Neurosyphilis	7	9.14months	10.8±3.9	CSF VDRL: positive in 5, sr.VDRL: positive in all

CSF: cerebrospinal fluid, HSV: herpes simplex virus, PCR: polymerase chain reaction, TBM: tubercular meningitis, HCP: hydrocephalus, ADA: adenosine deaminase, BAL: bronchoalveolar lavage, AFB: acid fast bacilli, SSPE: subacute sclerosing panencephalitis, VDRL: venereal disease research laboratory.

Of the immune mediated encephalitis/encephalopathy group, 20 patients (58.8%) had presented within 3 months of symptom onset, MRI brain was done in all the cases and showed typical bilateral medial temporal and hippocampal hyperintensities in 7 (20.5%) cases, while it was normal in 9 (26.4%) and showed non-specific changes not supportive for the diagnosis in the rest. Autoantibody panel was done in 21 (61.7%) patients, showed positive results in 4 (12.5%) patients. CSF analysis was done in 28 cases and was supportive for diagnosis showing elevated proteins and/or pleocytosis in 11(34.3%) patients. 6 cases had fulfilled criteria for definite autoimmune encephalitis (AIE) [4 antibody positive and 2 antibody negative AIE]. One case had systemic evidence of sarcoidosis (endobronchial biopsy positive) with no parenchymal brain involvement and responded to steroids and methotrexate.

Among the neurodegenerative group 9 (33.3%) had bvFTD, 6 (22.2%) were AD, 3 (11.1%) DLBD, 3 (11.1%) NPH, 2 (7.4%) CBS, 2 (7.4%) semantic variant FTD/PPA, 1 (3.7%) FTD-PSP and 1 (3.7%) FTD-P. PET supported diagnosis in 6 cases (4-bv-FTD, 1-AD and 1- DLBD).

Primary CNS lymphoma (n = 20) was the most common Neoplastic etiology presenting with RPD. FDG PET showed lesions with avid uptake in 7 cases. Brain biopsy was done in 10 cases with definite evidence of lymphoma in 8 and inconclusive findings in 2. Other causes included gliobastoma multiforme (autopsy-1 and imaging probable-1), gliomatosis cerebri (1), glioma (1) and metastatic CNS lymphoma (1) in a known case of Non-Hodgkin’s lymphoma.

Among vascular cognitive impairment and other cerebrovascular events group (n = 18) 3 patients had strategic infarcts, 12 multi-infarct states, 2 had chronic cerebral venous thromboses and one bilateral SDH. All except two patients underwent MRI with non-contrast CT head supporting diagnoses in the two patients with contraindications for MRI (metallic prostheses).

Among the prion disease group, all 14 cases satisfied the European criteria for probable sCJD. MR imaging was available in all 14 cases with 4 cases showing only cortical ribboning, 4 showing bilateral basal ganglia hyperintensities with diffusion restriction and 6 showing both. EEG was supportive for diagnosis with periodic discharges in 8 patients. None of our patients had underwent biopsy, CSF 14-3-3 or autopsy evaluations.

Primary CNS vasculitis comprised 10(5.3%) cases. Digital substraction angiography confirmed the diagnosis in 1 out of 4 cases. Dural based brain biopsy was done in 4 cases with features typical of granulomatous angitis seen in 3 cases and reactive gliosis in 1 case.

In patients with RPD due to nutritional and metabolic disorders alcohol related thiamine deficiency syndromes were the most common (with all 4 cases diagnosed on clinical and MRI findings). Hypothyroidism, non-ketotic hyperglycinemia and portal systemic encephalopathy were the rest.

## Associated neurological deficits

Other neurological deficits other than the cognitive domains were analysed (Table 3). Extrapyramidal signs (28.8%), seizures (21.3%), upper motor neuron features (19.7%), myoclonus (17.1%), cerebellar signs (13.9%) and vision loss (9.09%) were among the common associated features.

**Table 3:** Associated neurological deficits in patients with rapidly progressive dementia.

Sr.no	Category:		No of patients: n (%)
1	Vision loss		17 (9.09)
2	Other cranial nerve deficits		8 (4.2)
3	Pyramidal signs		37 (19.7)
4	Extrapyramidal signs and/or gait		54 (28.8)
5	Cerebellar signs		26 (13.9)
6	Small fiber neuropathy		8 (4.2)
7	Visual hallucinations		9 (4.8)
8	Generalized and/or focal seizures		40 (21.3)
9	Sleep disturbances	REM disorders	9 (4.8)
		Hypersomnolence	5 (2.6)
10	Hyperkinetic movement disorders:	Myoclonus	32 (17.1)
		Opsoclonus	4 (2.1)
		Choreo-athetosis	6 (3.1)
		Dystonia	12 (6.4)
		Dyskinesia	6 (3.1)

The clinical profile of patients in the secondary reversible, prion dementias and non-prion degenerative dementia groups is shown in Table 4.

**Table 4:** Comparison of secondary reversible, prion dementias and non-prion degenerative dementia categories.

Subgroup no.	Category:	No.	Mean age: years	Median age: Years	Sex F:M	Duration in months: Mean± SD (median)	Most common domain involved	MMSE Mean± SD	MMSE Median (IQR)
1	Reversible (treatable) secondary dementias:	126	44.6 ±18.6	47 (13–80)	48:78	4.44±3.83 (3)	Memory	17.41 ±6.12	18(12–22)
2	Prion dementias:	14	57.7±9.69	58.5 (44–71)	7:7	3.8±3.3(3)	Memory & inattention	19.54±1.85	20(18–22)
3	Non-prion Neurodegenerative and vascular dementias:	47	59.2±13.5	58(32–84)	9:37	7.9±3.9 (8)	Memory	15.29±5.88	16(10–20)
P value			0.0001^@^	0.0001^@^	0.045	0.0001^$^		0.039^ (One way ANOVA)	0.049^#^ (Kruskal Wallis test)

@Post hoc test with Bonferroni correction p value (1 vs 2) = 0.020; (2vs3) = 1.000; (1vs3) = 0.0001

$Post hoc test with Bonferroni correction p value (1 vs 2) = 1.000; (2vs3) = 0.002; (1vs3) = 0.0001 (The mean difference is significant at p< 0.017)

^ Post hoc test with Bonferroni correction for multiple comparisons (for all comparisons) statistically not significant at p<0.05

# Post hoc test with Bonferroni correction for multiple comparisons (for all comparisons) statistically not significant at p<0.05

The comparison of clinical and demographic profile in early (<6months) versus late (>6months) presentation is depicted in Table 5.

**Table 5:** Comparison of etiologic and investigation utilities in early versus late rapidly progressive dementia presentations.

Sr. no	Clinical feature:			No of patients (n)		P value
				< 6months (n = 129)	> 6 months (n = 58)	
1	Etiology	Nutritional		04	03	0.750
		Immune mediated		26	08	0.219
		Neoplastic		23	02	0.002
		Vascular		12	06	0.741
		Infectious		28	11	0.757
		Neurodegenerative:		09	18	0.000
		Pseudodementia:		04	01	1.000
		Prion diseases:		11	03	0.632
		PACNS		06	04	0.752
		Demyelinating disorders		05	01	0.790
		Dementia NOS:		01	01	0.701
2.	Cognitive domain involved:		Memory:	34	20	0.296
			Attention:	27	01	0.003
			Social cognition:	13	12	0.062
			Memory and attention:	19	05	0.345
			Memory and social cognition:	11	07	0.434
			Others:	25	13	0.695
3.	MRI in diagnosis:		Definite:	16	02	0.258
			Supportive:	57	27	
			Not contributory:	48	28	
4.	EEG in diagnosis:		Definite:	18	2	0.004
			Supportive:	4	1	
			Not contributory:	44	34	
5.	CSF analysis:	Raised proteins:	Supportive:	36	19	0.85
			Not contributory:	60	28	
		pleocytosis	Supportive:	18	5	0.332
			Not contributory:	78	42	

PACNS: primary CNS vasculitis, NOS: not otherwise specified MRI: magnetic resonance imaging, EEG: electroencephalography, CSF: cerebrospinal fluid. Using Fisher’s exact test.

## Investigation profile

### Serological tests

#### Autoimmune antibody panel

A panel for autoimmune antibodies [comprising: Anti VGKC (LG1, CASPR2), ANNA 1,ANNA 2, ANNA 3, AGNA, PCA 1, PCA 2, ANTI-Tr, AMPHIPHYSIN, CRMP5, Anti: Ma/Ta] was done in 21 patients, with positive results in 5 (anti-NMDA: 1, anti-Ma/Ta: 1, anti-purkinje cell antibody:1, anti-CASPR2: 1 and unclassified antibody of unknown significance: 1). Two patients underwent only NMDA antibody testing with positive result in one, while complete panel was done in rest.The panel was not tested due to financial constraints in 9 patients while results were not available in 3 patients.

#### Anti-TPO antibody test

Anti-TPO antibodies were tested in 140 patients and were elevated in 8 patients. Three of these had features typical of steroid responsive encephalopathy.

#### VDRL test

CSF-VDRL was done in 154 cases with positive results noted in 5 patients. Serum VDRL was done in 159 cases with positive results in 7 patients.

#### Serum B12

Serum B12 levels were tested in 32 cases, with below normal levels in 4.

#### Imaging

MRI was done in 178 cases and was supportive for diagnosis in 102 cases (73 early dementias vs. 29 late dementias; p = 0.258) with findings as described above in the respective etiologic subgroup. 16 cases had normal MRI, while 60 cases had non-specific changes (non specific T2 hyperintensities, diffuse cerebral atrophy, microangiopathic changes). Rest of the 9 patients with contraindications for MRI (metallic prosthesis) underwent non-contrast CT head.

#### CSF analysis

CSF evaluation was done in 143 cases and was supportive for diagnoses with raised proteins (n = 55; 38%) or pleocytosis (n = 23;16%). The details of findings have been described above.

#### EEG

EEG was performed in 103 cases and was supportive for diagnosis with periodic discharges in 20 cases (Prion: sJCD in 8 cases, SSPE:11 and HSV encephalitis:1)

## Discussion

We evaluated the clinico-epidemiological profile of patients with RPD admitted at our center over Jan2008-Aug 2016. In our study, secondary reversible causes formed the most common cause for RPD with infectious causes, immune mediated encephalitides and neoplastic disorders. This is in contrast to the evidence in literature that shows prion diseases as the leading cause of RPD in most case series which were based on prion disease and RPD referral centres [13,14]. Our study showed a younger onset of illness when compared to similar series reported earlier [15,16] and similar to the Studart Neto et al [17] series. This could be due to a larger proportion of infectious and autoimmune etiologies of RPDs which presented at younger age in our study population. (Table 6)

**Table 6:** Comparison between earlier RPD series and our study.

Study group:	No of cases (n)	Mean age at onset: (n± SD)	Median age (Range):	Symptom duration (median):	M:F	Most common etiology:
Papageorgiou et al.^15^ (Jan 2004-Dec 2006: RPD<1year)	68	65.5±10.0	66.7 (35.3–82.8)	6.7±3.06 (7)	37:31	1^st^: S-DEM: NPH 2^nd^: AD 3^rd^: FTD
Sala et al.^16^(Oct 1994-Mar 2009: RPD<1 year)	49	72.4±11.6	NA	4.6±3.8	22:27	1^st^ ND: AD 2^nd^: Prion disease 3^rd^: vascular and toxic/metabolic
Studart Neto et al^17^ (Mar 2012-Feb 2015: RPD<2years)	61	48±19.6	(14–84)	6.4±6.6	22:39	1^st^: immune mediated 2^nd^:infectious disorders 3^rd^: Prion diseases
Our study: (Jan 2008-Aug 2016: RPD<1 year)	187	49.3±18.2	50 (13–84)	5.28±4.1 (4)	122:65	1^st^: immune mediated 2^nd^: infectious disorders 3^rd^: non-prion neurodegenerative: FTD

SD: standard deviation, F: females, M: males, S-DEM: secondary dementia, NPH: normal pressure hydrocephalus, AD: Alzheimer’s disease, FTD: frontotemporal dementia, ND: neurodegenerative dementias.

Infections presenting with RPD accounted for the most common cause in our series (39%) with SSPE (41%) as the leading cause followed by neurosyphilis (17.9%) and PMLE (15.3%). NPDPSC [18] cohort reports infections to be fifth common cause for non-prion dementias, while neurosyphilis represented 2.9% cases in the RPD series by Papageorgiou et al.[15] Among patients with SSPE, 11(64.7%) were males, mean age of 19.7± 4.2 years and median age 20years (13–27 years). Past history of exanthematous fever was available in 10 (62.5%) cases. Only five (31.2%) patients had received immunization. Memory impairment and behavioral disturbances were most common cognitive domains involved and were the presenting features in 10 (62.5%) patients. Vision loss (4-posterior visual pathway; 2-maculopathy) was associated in 6 (37.5%) patients. Though none of the aforementioned RPD series or the young onset dementia series by Kelly et al. [19] had SSPE as a cause, there are case reports with SSPE presenting with dementia [20,21]. The higher proportion of cases with SSPE in our series could be due to improper immunisation, failure of cold chain maintenance or circulating atypical measles virus strain. Apart from the serological tests for HIV and other viral markers, serum and CSF VDRL test; EEG showing periodic discharges proved to be a useful aid in diagnosis in this category.

Immune mediated dementias formed the second most common (18.1%) etiologic cause for RPD. Although there was only one patient each of this group in earlier compared tertiary care centre series [15,16], it formed the second most common cause (13%) of non-prion RPD in the UCSF cohort [13]. These are characterized by rapidly progressive and fluctuating course, detection of inflammatory markers in CSF, presence of neural-specific antibodies and personal or family history or serologic evidence of other associated autoimmune conditions.[22] A trial of immunotherapy may serve as a diagnostic test. Presence of anti-neuronal antibodies is not required for diagnosis [22]. In our study the panel was tested for 21 patients and 4 revealed positive results. MRI showing bilateral medial temporal and hippocampal hyperintensities was supportive for diagnosis in 7 cases. CSF showing raised proteins and/or pleocytosis was supportive in 10 cases. All patients were treated with IV methyl prednisolone pulse, followed by oral steroids in 13 cases. Additional immunosuppression in the form of IVIg was given in 3 cases. Methotrexate, cyclophosphomide, mycophenolate and rituximab were given in one patient each. One patient had systemic sarcoidosis on CT chest and transbronchial lung biopsy, though Gad MRI brain did not reveal any lesions and responded well to steroid and methotrexate. Two patients were found to have neoplastic association (1- breast and 1-prostate) on systemic screening with FDG PET.

With increased awareness of this potentially treatable cause for cognitive decline and a possible sentinel marker of underlying undetected malignancy this entity is being increasingly searched for, therefore explaining the increasing proportion in newer series of RPD.

The neurodegenerative dementias were third common cause for RPD in our series while this was the most common cause in the Papageorgiou et al.[15] and Sala et al.[16] series. Among the non-prion degenerative group vascular cognitive impairment 18 (38.2%) and FTD behavioral and other variants (FTD-semantic variant-2, FTD-PSP-1 and FTD-P-1) were more common 13 (27.6%) followed by AD, DLBD, NPH,CBS and mixed dementia. Among other series AD was followed by FTD [14,23] while DLBD was reported to be more common cause for non-prion degenerative RPD in UCSF cohort [13].

Among the neoplastic causes, primary CNS lymphoma (n = 20) was the most common cause in our study group. With FDG PET showed lesions with avid uptake in 7 cases. Brain biopsy was done in 10 cases with definite evidence of lymphoma in 8 and inconclusive findings in 2. Other causes included gliobastoma multiforme (biopsy proven-1 and imaging probable-1), gliomatosis cerebri (1), glioma (1) and metastatic CNS lymphoma (1) in a known case of Non-Hodgkin’s lymphoma. Other series reported neoplastic causes to be third most common non-prion dementias (8%) in UCSF [13] series and fourth (8%) in NPDPSC [21] cohort.

Among patients with RPD due to nutritional and metabolic disorders alcohol related thiamine deficiency syndromes were the most common (with all 4 cases diagnosed on clinical and MRI findings). Hypothyroidism, non-ketotic hyperglycinemia and portal systemic encephalopathy were the rest. Alcohol related dementia may result from Wernicke–Korsakoff syndrome, Marchiafava–Bignami disease (MBD), hepatic encephalopathy, head injuries, subdural hematoma alone or in various combinations. Routine MRI or CT may help in diagnosis. Diffusion weighted imaging sequences may be required to establish subtle lesions in MBD. Prompt treatment with parenteral thiamine supplementation on clinical suspicion of thiamine deficiency is warranted.

Hypothyroidism is one of the most important causes of potentially reversible dementia.[24]The progressive neurocognitive decline seen in patients with untreated hypothyroidism can be averted or at least slowed with prompt and appropriate pharmacological management. Steroid responsive encephalopathy associated with autoimmune thyroiditis although not directly related to altered thyroid hormone status, is an important cause for reversible dementias. Our study included 3 cases with Hashimotos encephalopathy with steroid responsiveness, though Anti-TPO antibody levels were found to be elevated in 8 cases. The clinical presentation with fluctuating cognitive symptoms and myoclonus mimics sCJD. Hence thyroid function tests including anti-TPO antibody level form an indispensible part of RPD evaluation panel.

Hepatic or portal systemic encephalopathy can alter cognitive performance even without overt signs of hepatic failure [25]. Hyperammonemia may be central to its causation and the clinical state of the patient correlates well with arterial ammonia levels. Endogenous benzodiazepine ligands may also contribute. Electroencephalography (EEG) may show slow waves or triphasic waves. Increased signal intensity in the globus pallidus on T1-weighted MR images of the brain is typical and much of the cognitive dysfunction may improve with treatment.

Among prion disease group, all of our 14 cases met the European criteria [12] for diagnosis of probable sCJD. MRI and EEG aided in the diagnosis. Although none of our patients had undergone biopsy or CSF 14-3-3 analysis, previous studies evaluating accuracy of diagnostic criteria for sCJD [26] have shown high sensitivity combining DW images and CSF analysis with 14-3-3 and total tau protein level. Prion diseases have been reported to be the most common cause for RPD in UCSF cohort [13] while the second common cause in Sala et al [16] series.

Primary CNS vasculitis comprised 10(5.3%) cases. Digital substraction angiography confirmed the diagnosis in 1 out of 4 cases. Dural based brain biopsy was done in 4 cases with features typical of granulomatous angitis seen in 3 cases and reactive gliosis in 1 case. CNS vasculitis comprised 3% of the non-prion RPD cases in UCSF cohort [13].

We further subdivided the study group into early (≤6months) and late (>6 months) presentation. Among the etiologic profile, neoplastic disorders and immune mediated presented early while neurodegenerative disorders presented later.

**Fig 2:**
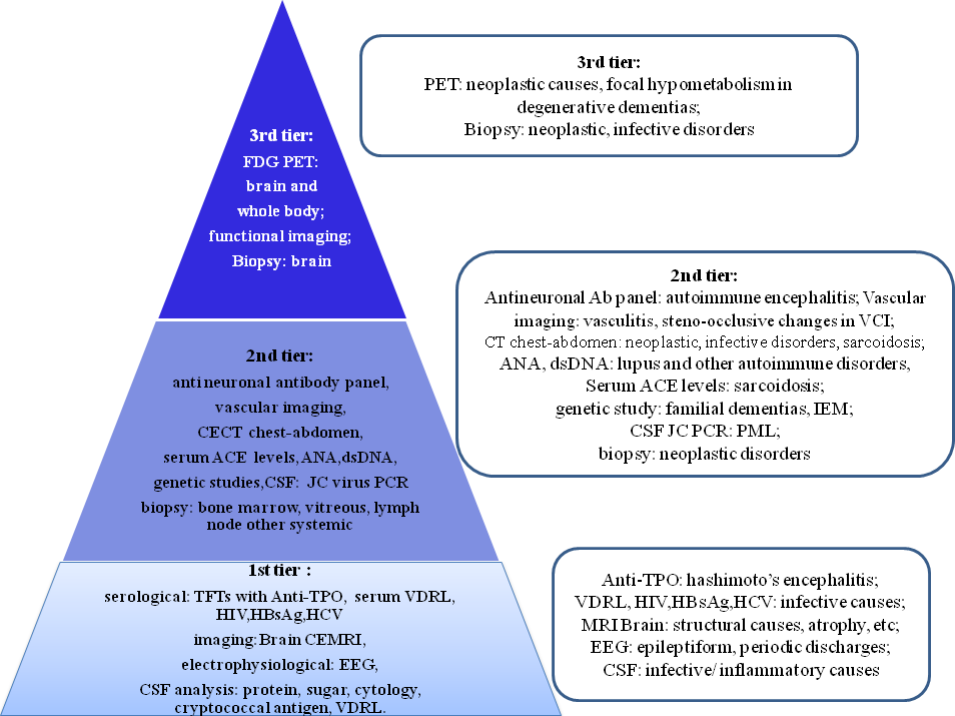
Investigation protocol for evaluation of rapidly progressive dementia. (TFT: thyroid function tests; Anti-TPO: anti thyroid peroxidase antibody, VDRL: veneral disease research laboratory test; CEMRI: contrast enhanced magnetic resonance imaging; EEG: electroencephalogram; ACE: angiotensin converting enzyme test; CECT: contrast enhanced computerised tomography; FDG PET: Fluorodeoxyglucose positron emission tomography scan; IEM: inborn errors of metabolism).

The possible etiologies that manifest as RPD are protean. It is prudent that the approach to RPD be directed at early recognition of reversible secondary causes. A detailed history and clinical examination is of paramount importance, which cannot be superseded by any investigations. We wish to propose a protocol approach to evaluating RPD (Fig 2).

### Limitations of the study

This was a retrospective study over 8 years (2008–2016). We had to rely on the records for clinical details and imaging reports. Individual MRI and CT scan films or computer based data were not available for assessment in patients before 2014. Detailed lobar function assessment and MMSE was routinely performed by the neurophysicians on duty and used for evaluation of all RPD patients. Thorough neuro-psychological assessment was not available for all the patients (except for the neurodegenerative dementia subgroup). Autoimmune antibody panel was done in 21 (62%) patients, (in 13 patients (38%) the panel could not be performed due to financial constraints, and were classified according to Graus et al criteria as antibody negative AIE or on the basis of response to immunotherapy as immune mediated dementias.)

FDG PET was not done in 142 cases (75.9%) due to financial constraints, it was a preferred investigation in immune-mediated dementia, Neoplastic and degenerative dementias. These findings were not individually used for statistical analysis.

Also few subgroups, as in infectious disorders with RPD had smaller sample size, inadequate for detailed statistical analysis.

### Conclusions

Rapidly progressive dementia is an emergency in cognitive neurology. We feel that many patients have treatable or reversible causes. Autoimmune encephalitis/ Immune mediated dementias, nutritional and infectious causes are the common causes of RPD in this study. A systematic evaluation can unravel treatable conditions that were doomed as degenerative entities in the past.

## Supporting information

S1 File Table A: Clinical and demographic profile of patients. Table B: infectious causes presenting with rapidly progressive dementia. Table C:Associated neurological deficits in patients with rapidly progressive dementia. Table D: Comparison of secondary reversible, prion dementias and non-prion degenerative dementia categories. Table E: Comparison of etiologic and investigation utilities in early versus late rapidly progressive dementia presentations. Table F: Comparison beween earlier RPD series and our study.
(PDF)

## References

[1] Geschwind MD. Rapidly progressive dementia: prion diseases and other rapid dementias. Continuum. 2010;16:31–56 doi: 10.1212/01.CON.0000368211.79211.4c 22810280 10.1212/01.CON.0000368211.79211.4c

[2] Paterson RW, Takada LT, Geschwind MD. Diagnosis and treatment of rapidly progressive dementias. Neurol Clin Pract. 2012; 2: 187–200. doi: 10.1212/CPJ.0b013e31826b2ae8 23634367 10.1212/CPJ.0b013e31826b2ae8 PMC3613204

[3] Degnan AJ, Levy LM. Neuroimaging of rapidly progressive dementias, part 1: neurodegenerative etiologies. Am J Neuroradiol. 2014;35:418–23. doi: 10.3174/ajnr.A3454 23436051 10.3174/ajnr.A3454 PMC7964711

[4] McKhann GM, Knopman DS, Chertkow H, Hyman BT, Jack CR Jr, Kawas CH et al The diagnosis of dementia due to Alzheimer's disease: recommendations from the National Institute on Aging-Alzheimer's Association workgroups on diagnostic guidelines for Alzheimer's disease. Alzheimers Dement. 2011;7:263–9. doi: 10.1016/j.jalz.2011.03.005 21514250 10.1016/j.jalz.2011.03.005 PMC3312024

[5] Roman GC, Tatemichi TK, Erkinjuntti T, Cummings JL, Masdeu JC, Garcia JH et al Vascular dementia: diagnostic criteria for research studies. Report of the NINDS-AIREN International Workshop. Neurology. 1993;43:250–260 8094895 10.1212/wnl.43.2.250

[6] McKeith IG, Galasko D, Kosaka K, Perry EK, Dickson DW, Hansen LA et al Consensus guidelines for the clinical and pathologic diagnosis of dementia with Lewy bodies (DLB): report of the consortium on DLB International Workshop. Neurology. 1996;47:1113–1124 8909416 10.1212/wnl.47.5.1113

[7] Rascovsky K, Hodges JR, Knopman D, Mendez MF, Kramer JH, Neuhaus J et al Sensitivity of revised diagnostic criteria for the behavioural variant of frontotemporal dementia. Brain. 2011;134:2456–77 doi: 10.1093/brain/awr179 21810890 10.1093/brain/awr179 PMC3170532

[8] Gorno-Tempini ML, Hillis AE, Weintraub S, Kertesz A, Mendez M, Cappa SF et al Classification of primary progressive aphasia and its variants. Neurology. 2011;76:1006–14. doi: 10.1212/WNL.0b013e31821103e6 21325651 10.1212/WNL.0b013e31821103e6 PMC3059138

[9] Graus F, Titulaer MJ, Balu R, Benseler S, Bien C, Cellucci T, et al A clinical approach to diagnosis of autoimmune encephalitis. Lancet Neurol. 2016 4;15(4):3991–404. 10.1016/S1474-4422(15)00401-9 PMC5066574 26906964

[10] Dyken P.R. Subacute sclerosing panencephalitis. Neurol clin 1985;3:179–95. 2581121

[11] Zerr I, Kallenberg K, Summers DM, Romero C, Taratuto A, Heinemann U et al Updated clinical diagnostic criteria for sporadic Creutzfeldt-Jakob disease. Brain. 2009;132:2659–68. doi: 10.1093/brain/awp191 19773352 10.1093/brain/awp191 PMC2759336

[12] Calabrese L.H., Mallek J.A. Primary angiitis of the central nervous system. Report of 8 new cases, review of the literature, and proposal for diagnostic criteria. Medicine (Baltimore). 1988;67:20–39. 3275856 10.1097/00005792-198801000-00002

[13] Geschwind MD. Rapidly progressive dementia: prion diseases and other rapid dementias. Continuum. 2016;22(2):510–37. 22810280 10.1212/01.CON.0000368211.79211.4c

[14] Grau-Rivera O, Gelpi E, Nos C, Gaig C, Ferrer I, Saiz A, et al Clinicopathological correlations and concomitant pathologies in Rapidly Progressive Dementia: A Brain Bank Series. Neurodegener Dis. 2015;15(6):350–60. doi: 10.1159/000439251 26523804 10.1159/000439251

[15] Papageorgiou S.G, Kontaxis T., Bonakis A, Karahalios G, Kalfakis N, Vassilopoulos D. Rapidly Progressive Dementia Causes Found in a Greek Tertiary Referral Center in Athens. Alzheimer Dis Assoc Disord. 2009;23:337–46. doi: 10.1097/WAD.0b013e31819e099b 19561440 10.1097/WAD.0b013e31819e099b

[16] Sala I, Marquié M, Sánchez-Saudinós MB, Sánchez-Valle R, Alcolea D, Gómez-Ansón B et al Rapidly progressive dementia: experience in a tertiary care medical center. Alzheimer Dis Assoc Disord. 2012;26:267–71. doi: 10.1097/WAD.0b013e3182368ed4 22001379 10.1097/WAD.0b013e3182368ed4

[17] Studart Neto A, Soares Neto H, Simabukuro M, Solla D, Goncalves M, Fortini I, et al Rapidly progressive dementia: Prevalence and causes in a neurologic unit of tertiary hospital in Brazil. Alzheimer Dis Assoc Disord.2017;31(3):239–43. doi: 10.1097/WAD.0000000000000170 27849640 10.1097/WAD.0000000000000170

[18] Chitravas N., Jung R.S., Kofskey D.M, Blevins JE, Gambetti P, Leigh RJ et al Treatable Neurological Disorders Misdiagnosed as Creutzfeldt—Jakob disease. Ann Neurol. 2011; 70: 437–444. doi: 10.1002/ana.22454 21674591 10.1002/ana.22454 PMC3170496

[19] Kelley BJ, Boeve BF, Josephs KA. Young-onset dementia: demographic and etiologic characteristics of 235 patients. Arch Neurol 2008; 65: 1502–8. doi: 10.1001/archneur.65.11.1502 19001170 10.1001/archneur.65.11.1502

[20] Chakor R.T., Santosh N.S. Subacute sclerosing panencephalitis presenting as rapidly progressive young-onset dementia. Journal of the Pakistan Medical Association 2013; 63: 921–4. 23901723

[21] Singer C, Lang AE, Suchowersky O. Adult-onset subacute sclerosing panencephalitis: case reports and review of the literature. Mov Disord. 1997;12:342–53. doi: 10.1002/mds.870120313 9159729 10.1002/mds.870120313

[22] McKeon A, Lennon VA, Pittock SJ. Immunotherapy-responsive dementias and encephalopathies. Continuum. 2010;16:80–101. doi: 10.1212/01.CON.0000368213.63964.34 22810282 10.1212/01.CON.0000368213.63964.34

[23] Poser S., Mollenhauer B., Kraubeta A, Zerr I, Steinhoff BJ, Schroeter A et al How to improve the clinical diagnosis of Creutzfeldt–Jakob disease. Brian 1999; 122:2345–51. 10.1093/brain/122.12.2345 10581227

[24] Dugbartey A.T. Neurocognitive Aspects of Hypothyroidism. Arch Intern Med. 1998;158:1413–1418. 9665349 10.1001/archinte.158.13.1413

[25] Gilberstadt SJ, Gilberstadt H, Zieve L, Buegel B, Collier RO Jr, McClain CJ. Psychomotor performance defects in cirrhotic patients without overt encephalopathy. Arch Intern Med 1980;140:519–21. 7362383

[26] Tagliapietra M, Zanusso G, Fiorini M, Bonetto N, Zarantonello G, Zambon A, et al Accuracy of diagnostic criteria for sporadic creutzfeldt-jakob disease among rapidly progressive dementia. J Alzheimers Dis.2013;34(1):231–8. doi: 10.3233/JAD-121873 23207489 10.3233/JAD-121873

