# Supplementary material for: Rapidly progressive dementia: An eight year (2008–2016) retrospective study
Source: PLoS One. 2018 Jan 18;13(1):e0189832. doi: 10.1371/journal.pone.0189832 (PMC5773088; doi:10.1371/journal.pone.0189832)
Supplement: S1 File — Table A: Clinical and demographic profile of patients. Table B: infectious causes presenting with rapidly progressive dementia. Table C:Associated neurological deficits in patients with rapidly progressive dementia. Table D: Comparison of secondary reversible, prion dementias and non-prion degenerative dementia categories. Table E: Comparison of etiologic and investigation utilities in early versus late rapidly progressive dementia presentations. Table F: Comparison beween earlier RPD series and our study. (PDF) [file pone.0189832.s001.pdf]

Table A in S1 file: clinical and demographic profile of patients as per the etiologic subgroups

Sr no.	Etiological subgroup	No (n)	Mean age±SD years	Median age (IQR) years	Males (%)	duration in months Mean ± SD (median)	Average MMSE score (mean±SD)
1	Infectious disorders	39	32.4±16.8	25(20-42)	26(81.2%)	5.9±3.9(4)	18.14±6.09
2	Immune mediated encephalitis/encephalopathy	34	54.5±16.9	55 (45,69.2)	16(50%)	3.9±3.8 (2)	18.15±7.07
3	Neurodegenerative disorders	27	56.2±13.3	58(43.5-65.5)	21(84%)	9.2±3.9(12)	18.6±5.83
4	Neoplastic or metastatic disorders	25	56.17±14.1	58 (47.5-60.5)	15(65.2%)	3.04±2.6 (1)	18.8±7.71
5	Vascular cognitive imparment and other cerebrovascular events:	18	62.3±13.7	59 (50.7-78)	13(81.2%)	6.7±3.4(6)	11.4±3.5
6	Prion diseases	14	57.7±9.6	58(47.7-67)	7(50%)	3.8±3.3(3)	20.5±2.06
7	PACNS:	10	35±9.9	35.5(30.5-41)	8(80%)	5.9±4.4(5.5)	22.3±6.28
8	Nutritional and metabolic disorders	7	42.14±15.1	50 (25-54)	5(83.3%)	6.3± 4.8(8)	18.25±5.9
9	Demyelinating disorders:	6	38.5±17.7	39.5(21-51.5)	3(50%)	3.33±3.5(2.25)	24.6±2.64

10	Psychiatric Conditions:	5	56.8±13.1	64(42.5-67.5)	1(20%)	5±4.3(4)	28.2±1.3
11	Mixed / undetermined dementia	2	53.3±20.9	53	6(60%)	5.2±4.02(4.5)	22±2

Table B in S1 file: Infectious causes presenting with rapidly progressive dementia:

Sr. no	Diagnosis:	No. of patients:	Symptom duration:	MMSE (n±SD)	Investigations:
1	HSV encephalitis	1	15days	8	CSF HSV PCR: positive
2	Tubercular meningitis	2	5.5months	19.12	CSF: normal; communicating HCP with ring enhancing granulomas in parietal lobe.
3	Cryptococcal meningitis	1	4months	21	Cryptococcal antigen titre-1:64
4	TBM+ Cryptococcal meningitis	1	12months	17	CSF proteins:110, sugar:10, ADA:16, cryptococcal culture +; BAL: AFB2+
5	Neurocysticercosis	3	3.3months	17	Not done: 2; Normal:

					1
6	SSPE	17	3.94months	17.5±7.7	Raised CSF anti-measles antibody titre: 9; normal titre:1; not done: 6
7	HIV dementia	1	3months	24	CSF: not done
8	HIV + PMLE	6	4.33months	15±1.41	Mean CD4 count:95;
9	Neurosyphilis	7	9.14months	10.8±3.9	CSF VDRL: positive in 5, sr.VDRL: positive in all

Table C in S1 file: Associated neurological deficits in patients with rapidly progressive dementia

Sr.no	Category:	No of patients: n (%)
1	Vision loss	17 (9.09)
2	Other cranial nerve deficits	8 (4.2)
3	Pyramidal signs	37 (19.7)
4	Extrapyramidal signs and/or gait	54 (28.8)
5	Cerebellar signs	26 (13.9)
6	Small fiber neuropathy	8 (4.2)
7	Visual hallucinations	9 (4.8)
8	Generalized and/or focal seizures	40 (21.3)

9	Sleep disturbances	REM disorders	9 (4.8)
		Hypersomnolence	5 (2.6)
10	Hyperkinetic movement disorders:	Myoclonus	32 (17.1)
		Opsoclonus	4 (2.1)
		Choreo-athetosis	6 (3.1)
		Dystonia	12 (6.4)
		Dyskinesia	6 (3.1)

Table D in S1 file: comparison of secondary reversible, prion dementias and non-prion degenerative dementia categories

Subgroup no.	Category:	No.	Mean age: years	Median age: Years	Sex F:M	Duration in months: Mean± SD (median)	Most common domain involved	MMSE Mean± SD	MMSE Median (IQR)
1	Reversible (treatable) secondary dementias:	126	44.6 ±18.6	47 (13-80)	48:78	4.44±3.83 (3)	Memory	17.41 ±6.12	18(12-22)

2	Prion dementias:	14	57.7±9.69	58.5 (44-71)	7:7	3.8±3.3(3)	Memory & inattention	19.54±1.85	20(18-22)
3	Non-prion Neurodegenerative and vascular dementias:	47	59.2±13.5	58(32-84)	9:37	7.9±3.9 (8)	Memory	15.29±5.88	16(10-20)
P value			0.0001[@]	0.0001[@]	0.045	0.0001[$]		0.039^ (One way ANOVA)	0.049[#] (Kruskal Wallis test)

Table E in S1 file: Comparison of etiologic and investigation utilities in early versus late Rapidly Progressive Dementia presentations

Sr. no	Clinical feature:		No of patients (n)		P value
			< 6months (n=129)	> 6 months (n=58)	
1	Etiology	Nutritional	04	03	0.750

		Immune mediated	26	08	0.219
		Neoplastic	23	02	0.002
		Vascular	12	06	0.741
		Infectious	28	11	0.757
		Neurodegenerative:	09	18	0.000
		Pseudodementia:	04	01	1.000
		Prion diseases:	11	03	0.632
		PACNS	06	04	0.752
		Demyelinating disorders	05	01	0.790
		Dementia NOS:	01	01	0.701
2.	Cognitive domain involved:	Memory:	34	20	0.296
		Attention:	27	01	0.003
		Social cognition:	13	12	0.062
		Memory and attention:	19	05	0.345
		Memory and social cognition:	11	07	0.434
		Others:	25	13	0.695
3.	MRI in diagnosis:	Definite:	16	02	0.258
		Supportive:	57	27	
		Not contributory:	48	28	
4.	EEG in diagnosis:	Definite:	18	2	0.004
		Supportive:	4	1	

			Not contributory:	44	34	
5.	CSF analysis:	Raised proteins:	Supportive:	36	19	0.85
			Not contributory:	60	28	
		pleocytosis	Supportive:	18	5	0.332
			Not contributory:	78	42	

Table F in S1 file: comparison between earlier RPD series and our study:

Study group:	No of cases (n)	Mean age at onset: (n± SD)	Median age (Range):	Symptom duration (median):	M:F	Most common etiology:
Papageorgiou et al.[15] (Jan 2004-Dec 2006: RPD<1year)	68	65.5±10.0	66.7 (35.3-82.8)	6.7±3.06 (7)	37:31	1st: S-DEM: NPH<br>2nd: AD<br>3rd: FTD
Sala et al.[16](Oct 1994-Mar 2009: RPD<1 year)	49	72.4±11.6	NA	4.6±3.8	22:27	1st ND: AD<br>2nd: Prion disease<br>3rd: vascular and toxic/metabolic
Studart Neto et al[17] (Mar 2012-Feb 2015: RPD<2years)	61	48±19.6	(14-84)	6.4±6.6	22:39	1st: immune mediated<br>2nd:infectious disorders<br>3rd: Prion diseases

Our study: (Jan 2008-Aug 2016: RPD<1 year)	187	49.3±18.2	50 (13-84)	5.28±4.1 (4)	122:65	1st: immune mediated<br>2nd: infectious disorders<br>3rd: non-prion neurodegenerative: FTD
